# Stunting Among Under 5-Year-Olds in Nepal: Trends and Risk Factors

**DOI:** 10.1007/s10995-019-02817-1

**Published:** 2019-11-27

**Authors:** Shyam Sundar Budhathoki, Amit Bhandari, Rejina Gurung, Abhishek Gurung, Ashish KC

**Affiliations:** 1 Golden Community, Lalitpur, Nepal; 2 School of Public Health and Community Medicine, B.P. Koirala Institute of Health Sciences, Dharan, Nepal; 3 Society of Public Health Physicians Nepal, Kathmandu, Nepal; 4 International Maternal and Child Health, Department of Women’s and Children’s Health, University Hospital, Uppsala University, 751 85 Uppsala, Sweden

**Keywords:** Stunting, Multi-dimensional risk factors, Nepal, Sustainable development goals, Early childhood development, Undernutrition

## Abstract

**Introduction:**

The nutritional status in the first 5 years of life has lifelong and inter-generational impacts on individual’s potential and development. This study described the trend of stunting and its risk factors in children under 5 years of age between 2001 and 2016 in Nepal.

**Methods:**

The study used datasets from the 2001, 2006, 2011 and 2016 Nepal Demographic Health Surveys to describe the trend of stunting in under 5-year children. Multiple logistic regression analysis was carried out to assess the risk factors for stunting at the time of the four surveys.

**Results:**

The nutritional status of under 5-year children improved between 2001 and 2016. Babies born into poorer families had a higher risk of stunting than those born into wealthier families (AOR 1.51, CI 95% 1.23–1.87). Families residing in hill districts had less risk of stunting than those in the Terai plains (AOR 0.75, CI 95% 0.61–0.94). Babies born to uneducated women had a higher risk of stunting than those born to educated women (AOR 1.57, CI 95% 1.28–1.92).

**Discussion:**

Stunting among under-5-year children decreased in the years spanning 2001–2016. This study demonstrated multiple factors that can be addressed to decrease the risk of stunting, which has important implications for neurodevelopment later in life. We add literature on risk factors for stunting in under-5-year children.

## Significance

Improving the nutritional status of women and children is key for achieving many of the Sustainable Development Goals as proper nutrition underlies the development and potential of the population. Stunting in young children is caused by a range of risk factors. This paper explores the trend in the stunting in children. This study elucidates risk factors associated with stunting.

## Introduction

The nutritional status of a child in his/her first 5 years of life is correlated with its linear growth, its cognitive development and the prevention of chronic diseases later in life (Adair et al. 2013; Barker 2012; Black et al. 2008). Adequate growth and development in early childhood primarily depends on nutrition, and thus investment in improved nutrition is an essential foundation for the development of human capital (Lu et al. 2016). Yet, in 2011, 32.4 million babies were born small for their gestational age with 27% of these babies born in low and middle-income countries (Lee et al. 2013).

There are many factors associated with stunting, defined as children with their length/height below 2 standard deviations from the WHO Child Growth Standards median for same age and sex (WHO 2006). Social factors such as poverty, place of residence and ethnicity are associated with children’s nutritional status (Black et al. 2008, 2013; Horton and Lo 2013; Ikeda et al. 2013; Katz et al. 2013; Lee et al. 2013; Lu et al. 2016; Richter et al. 2017; United Nations 2016). Environmental and behavioural factors, such as l mother’s lack of education, lack of hygiene, poor sanitation and indoor air pollution also increase the risk of stunting in young children (Kismul et al. 2018). Health interventions such as disease prevention and the early diagnosis and treatment of disease protect against stunting (Kuhnt and Vollmer 2017). Reducing the various risk factors through multi-sectoral interventions will prevent the inter-generational cycle of poor nutrition (Barker et al. 1993; Bhutta 2013).

The Millennium Development Goals (MDGs 2000–2015) helped establish nutrition as a key factor on the global and multi-sectoral agenda for development (The World Bank 2006). MDG 1, which aimed to reduce poverty by half between 2000 and 2015, triggered the need to have nutrition-sensitive interventions to reduce malnutrition. MDGs 4 and 5 aimed to reduce under-5-year and maternal mortality through implementation of health-related interventions to prevent and manage malnutrition for women and children. As a result, United Nations agencies initiated the Scaling Up Nutrition and the Global Alliance Advocacy and Agenda to raise the profile and increase level of investment in nutrition (Bezanson and Isenman 2010; Pearson and Ljungqvist 2011; Victora et al. 2012). As a result, multi-sectoral nutrition plans were developed and implemented globally, including in Nepal. The SDG agenda expanded upon this by setting nutrition as a primary pathway for early childhood development (Barros and Ewerling 2016; United Nations 2015).

Nepal made good progress on reducing undernutrition in under 5-year during the MDG era (Ministry of Health and Population, Nepal 2017). The underweight in under-5-year children decreased from 43% in 2001 to 27% in 2016 while stunting decreased from 57% in 2001 to 36% in 2016 (Ministry of Health and Population, Nepal 2017). Based on the Nutritional Assessment and Gap Analysis (NAGA) report, a Multi-Sectoral Nutrition Plan (MSNP) was developed through inter-ministerial consultations for the 2013–2017 period (National Planning Commission 2012; Pokharel et al. 2009). The plan was based on reducing the risk factors for undernutrition related to health, nutrition, water, sanitation, education, poverty reduction and food production (National Planning Commission, Nepal 2012).

This paper examines the trends and risk factors for stunting in under 5-year children in Nepal between 2001 and 2016.

## Methods

This study used secondary data from the 2001, 2006, 2011 and 2016 Nepal Demographic Health Surveys (NDHSs) datasets (Ministry of Health & Population, Nepal 2002, Ministry of Health, Nepal 2007, Ministry of Health and Population, Nepal 2012, Ministry of Health, Nepal 2017). These every five surveys are cross-sectional nationwide surveys carried out using standard methods. Household level interviews are conducted with men and women in order to gather information on the socioeconomic, health and nutritional status of families.

The 2001 NDHS interviewed 8400 women (aged 15–49 years), the 2006 NDHS 8600 women, the 2011 NDHS, 13,485 women and the 2016 NDHS, 13,089 women across 13 or 14 domains based on the most recent completed census. The women’s questionnaires had a 98–99% response rate.

### Data Collection

The women’s questionnaires collected information from women aged 15–49 years. They were asked about their level of education, place of residence, exposure to the media, pregnancy history, childhood mortality, breastfeeding and infant feeding practices, childhood immunizations and illnesses, marital status, sexual activity, work status, their husband’s background characteristics, awareness and behaviour related to HIV/AIDS and other sexual transmitted infections, and maternal mortality.

### Data Management

The current study extracted all the relevant data from the four surveys into SPSS version 23. The data were weighted using the sample weights of each individual sample. The variables with multiple categories were recoded into dichotomous categories.

The study defined stunting as “children with their length/height below 2 standard deviations from the WHO Child Growth Standards median for same age and sex” and considered stunting as the outcome indicator for analysis (WHO 2006).

The following variables, grouped by factor, were extracted from the NDHS datasets to assess the association of the risk factors with stunting:

- Socio-demographic factors
  1. Wealth quintile—Wealth quintile was assessed by collecting data regarding possession of durable assets (e.g. radios, televisions, refrigerators, cars, bicycles), housing characteristics (e.g. toilet facilities, number of rooms, materials used for roof and floor), and accessibility to services (e.g. source of drinking water and electricity supply). A wealth index using the scores was created from the first analysis of principal component and individuals were sorted in population quintiles from poorest to richest (Bhutta et al. 2013; Filmer and Pritchett 2001).
  2. Ecological region-mountain, hills and Terai,
  3. Urban or rural residence.
  4. Main type of cooking fuel—Either clean fuel (electricity, LPG [liquefied petroleum gas], biogas, natural gas), or polluted fuel (kerosene, coal, charcoal, wood, straw, shrubs, grass, animal dung).
  5. Toilet facility in home.
- Maternal factor
  6. Maternal (formal) education level—‘Educated’ if had at least completed primary education and vice versa.
  7. Maternal occupation—Mother in paid employment or not.
  8. Occurrence of indoor tobacco smoking.
- Obstetric factor
  9. Mother having attended four or more or less antenatal (ANC) care visits.
  10. Mother took iron supplementation during pregnancy.
  11. Delivery took place at home or in a health institution.
- Neonatal factor
  12. Sex of the child
  13. Size at birth
  14. Baby ever or never breastfed.

### Data Analysis

All statistical analysis was done using SPSS version 23. The study described the trend of the under-5-year stunting between 2001 to 2016 using the four datasets. Tests of association between stunting and socio-demographic factors were carried out using Pearson’s Chi square test and student *t* test.

Variables with a p value of < 0.1 were considered for multi-variable logistic regression. The variables were dichotomized for this analysis. Factors such as wealth, place of residence, maternal education and sex were assessed for collinearity. Those showing collinearity were not included in the multi-variable analysis.

The wealth quintiles were dichotomized into poor (poorest to middle quintile) and rich (richest and richer quintiles) while antenatal care was categorized into more than equal to 4 visits, less than 4 visits and no visits.

## Results

**Fig. 1:**
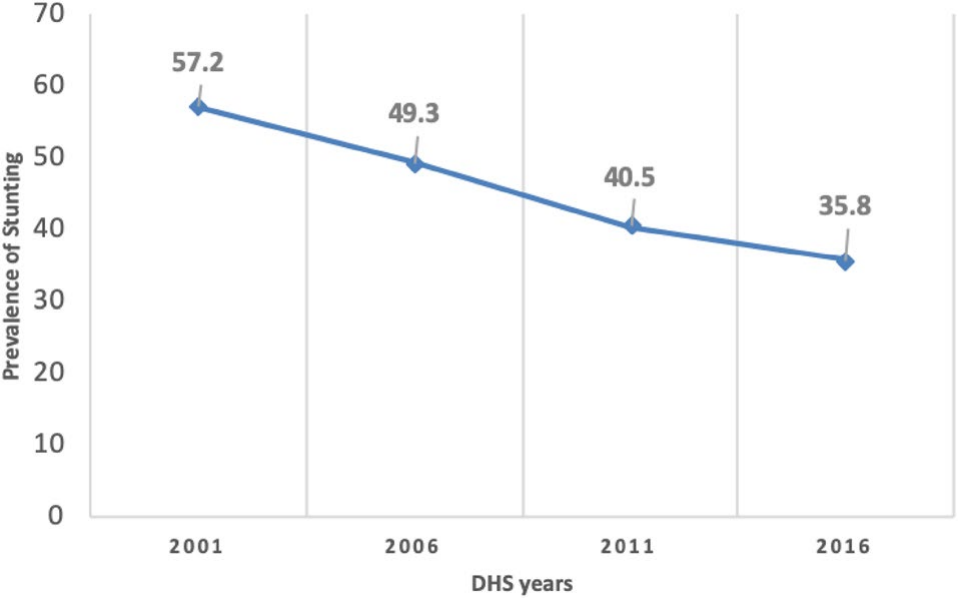
Stunting in under 5-year old in the 2001–2016 NDHSs

The proportion of stunted five-year olds in Nepal decreased by 21 percentage points between 2001 and 2016 (Fig. 1). There were significant associations (p < 0.01) between stunting and the following 12 factors in the 2001, 2006, 2011 and 2016 datasets—economic status, ecological region, place of residence, maternal education, working status of mothers, type of cooking fuel, toilet facilities at home, indoor smoking, antenatal visit, iron supplementation during pregnancy, health institution delivery and small size at birth (Table 1).

**Table 1:** Association between socio-demographic, health service and biological factors with stunting in under 5 year olds in 2001, 2006, 2011 and 2016 (NDHS data)

Characters	2001		p value	2006		p value	2011		p value	2016		p value
	No (%)	Yes (%)		No (%)	Yes (%)		No (%)	Yes (%)		No (%)	Yes (%)	
Sex												
Male	1616 (51.6)	1513 (48.4)	0.04	1316 (51.2)	1253 (48.8)	0.51	710 (58.8)	497 (41.2)	0.35	796 (64.3)	442 (35.7)	0.984
Female	1563 (49.1)	121 (50.9)		1235 (50.3)	1221 (49.7)		712 (60.7)	461 (39.3)		727 (64.3)	403 (35.7)	
Total	3179 (50.4)	3134 (49.6)		2551 (50.8)	2474 (49.2)		1422 (59.7)	958 (40.3)		1523 (64.3)	845 (35.7)	
Wealth index												
Poorest				492 (38.7)	779 (61.3)	< 0.01	267 (43.8)	342 (56.2)	< 0.01	249 (51.0)	239 (49.0)	< 0.01
Poorer				491 (45.5)	587 (54.4)		264 (54.5)	219 (45.3)		318 (61.9)	196 (38.1)	
Middle				500 (49.3)	515 (50.7)		361 (65.0)	(35.0)		347 (64.7)	189 (35.5)	
Richer				543 (59.9)	367 (40.1)		285 (70.0)	122 (30.0)		346 (67.6)	166 (32.4)	
Richest				519 (69.8)	225 (30.2)		246 (75.0)	82 (25.0)		263 (82.4)	56 (17.6)	
Total				2551 (50.8)	959 (40.3)		1423 (59.7)	959 (40.3)		1523 (64.3)	846 (35.7)	
Ecological region												
Mountain	183 (38.9)	287 (61.1)	< 0.01	165 (38.7)	261 (61.3)	< 0.01	88 (46.8)	100 (53.2)	< 0.01	89 (53.6)	77 (46.4)	< 0.01
Hill	1277 (48.3)	1366 (51.7)		1031 (49.8)	1040 (50.2)		548 (58.3)	392 (41.7)		587 (67.4)	284 (32.6)	
Terai	1719 (53.7)	1481 (46.3)		1355 (53.6)	1173 (46.4)		786 (62.8)	466 (37.2)		847 (63.6)	485 (36.4)	
Total	3179 (50.4)	3134 (49.6)		2551 (50.8)	2474 (49.2)		1523 (64.3)	846 (35.7)		1523 (64.3)	846 (35.7)	
Place of residence												
Urban	262 (63.7)	149 (36.3)	< 0.01	394 (64.6)	216 (35.4)	< 0.01	154 (73.0)	57 (27.0)	< 0.01	855 (68.2)	399 (31.8)	< 0.01
Rural	2808 (48.5)	2985 (51.5)		2156 (48.8)	2258 (51.2)		1268 (58.5)	901 (41.5)		668 (59.9)	447 (40.1)	
Total	3070 (49.5)	3134 (50.5)		2250 (50.8)	2474 (49.2)		1422 (59.7)	958 (40.3)		1523 (64.3)	846 (35.7)	
Maternal education												
Uneducated	2061 (45.1)	2507 (54.9)	< 0.01	1271 (42.3)	1735 (57.7)	< 0.01	593 (52.5)	536 (47.5)	<0.01	444 (54.3)	374 (45.7)	<0.01
Educated	1009 (61.7)	627 (38.3)		1279 (63.4)	739 (36.6)		829 (66.2)	423 (33.8)		1079 (69.6)	472 (30.4)	
Total	3070 (49.5)	3134 (50.5)		2550 (50.8)	2474 (49.2)		1422 (59.7)	959 (40.3)		1523 (64.3)	846 (35.7)	
Work status of mother												
No	597 (58.6)	421 (41.4)	< 0.01	586 (59.4)	401 (40.6)	< 0.01	446 (66.7)	223 (33.3)	< 0.01	659 (70.1)	281 (29.1)	< 0.01
Yes	2472 (47.7)	2721 (52.3)		1965 (48.7)	2073 (51.3)		976 (57.0)	735 (43.0)		864 (60.5)	565 (39.5)	
Total	3069 (49.5)	3133 (50.5)		2551 (50.8)	2474 (49.2)		1422 (59.7)	958 (40.3)		1523 (64.3)	846 (39.5)	
Cooking fuel												
Polluting	2674 (48.2)	2272 (52.1)	< 0.01	2091 (47.9)	2272 (52.1)	< 0.01	1072 (56.2)	835 (43.8)	< 0.01	403 (77.1)	120 (22.9)	< 0.01
Clean	144 (69.6)	63 (30.4)		284 (76.1)	89 (23.9)		237 (74.5)	81 (25.5)		1002 (59.7)	675 (36.1)	
Total	2818 (48.9)	2942 (51.1)		2375 (50.1)	2361 (49.9)		1309 (58.8)	916 (41.2)		1405 (63.9)	795 (36.1)	
Toilet facilities in home												
No	2063 (45.6)	2462 (54.4)	< 0.01	1135 (60.1)	752 (39.9)	< 0.01	723 (64.1)	405 (35.9)	< 0.01	272 (52.4)	247 (47.6)	< 0.01
Yes	796 (59.5)	542 (40.5)		1239 (43.5)	1612 (56.5)		586 (53.4)	512 (46.6)		1133 (67.4)	548 (32.6)	
Total	2859 (48.8)	3004 (51.2)		2374 (50.1)	2364 (49.9)		1309 (58.8)	917 (41.2)		1405 (63.9)	795 (36.1)	
Indoor tobacco smoking												
No	654 (40.4)	963 (59.6)	< 0.01	352 (36.5)	613 (63.5)	< 0.01	136 (44.6)	169 (55.4)	< 0.01	70 (53.0)	62 (47.0)	< 0.01
Yes	2416 (52.7)	2171 (47.3)		2199 (54.2)	1861 (45.8)		1286 (62.0)	789 (38.0)		1453 (65.0)	784 (35.0)	
Total	3070 (49.5)	3134 (50.5)		2551 (50.8)	2474 (49.2)		1422 (59.3)	958 (40.3)		1523 (64.3)	846 (35.7)	
Antenatal care visits												
Less than 4	1967 (52.5)	1783 (47.5)	< 0.01	1315 (49.8)	1328 (50.2)	< 0.01	556 (57.7)	407 (42.3)	< 0.01	351 (58.4)	250 (41.6)	< 0.01
≥4	445 (70.1)	190 (29.9)		795 (69.7)	345 (30.3)		644 (69.9)	277 (30.1)		949 (71.1)	385 (28.9)	
Total	2412 (55.0)	1973 (45.0)		2110 (55.8)	1673 (44.2)		1200 (63.7)	684 (36.3)		1300 (67.2)	635 (32.8)	
Iron supplementation during pregnancy												
No	1785 (52.2)	1635 (47.8)	< 0.01	698 (45.8)	822 (54.2)	< 0.01	210 (53.6)	182 (46.4)	< 0.01	110 (58.8)	77 (41.2)	0.01
Yes	682 (66.9)	338 (33.1)		1415 (62.4)	851 (37.6)		990 (66.4)	502 (33.6)		1190 (68.1)	558 (31.9)	
Total	2467 (55.6)	1973 (44.4)		2109 (55.8)	1673 (44.2)		1200 (63.7)	684 (36.3)		1300 (67.2)	635 (32.8)	
Place of delivery												
Home	2629 (47.8)	2872 (52.2)	< 0.01	1904 (46.6)	2180 (53.4)	< 0.01	796 (52.2)	728 (47.8)	< 0.01	543 (55.5)	435 (44.5)	< 0.01
Health institution	382 (66.6)	192 (33.4)		619 (70.6)	258 (29.4)		603 (73.5)	217 (26.5)		911 (70.8)	376 (29.2)	
Total	3011 (49.6)	3064 (50.4)		2523 (50.9)	2438 (49.1)		1399 (59.7)	945 (40.3)		1454 (64.2)	811 (35.8)	
Postnatal check up												
Skilled worker	115 (58.4)	82 (41.6)	0.78	131 (57.2)	98 (42.8)	0.06	547 (71.8)	215 (28.2)	< 0.01	433 (71.3)	174 (28.7)	0.08
Non-skilled worker	23 (63.9)	13 (36.1)		28 (57.1)	21 (42.9)		91 (58.7)	64 (41.3)		51 (60.7)	33 (39.3)	
Traditional/other	333 (57.9)	242 (42.1)		40 (58.8)	28 (41.2)		2 (66.7)	1 (33.3)		10 (83.3)	2 (16.7)	
Total	471 (58.3)	337 (41.7)		199 (57.5)	147 (42.5)		640 (69.6)	280 (30.4)		494 (70.3)	209 (29.7)	
Small size at birth												
No	2664 (53.1)	2351 (46.9)	< 0.01	2173 (53.1)	1919 (46.9)	< 0.01	1212 (62.0)	743 (38.0)	< 0.01	1302 (66.2)	666 (33.2)	< 0.01
Yes	514 (39.6)	783 (60.4)		376 (40.6)	553 (59.4)		207 (49.3)	213 (50.7)		218 (55.1)	178 (44.9)	
Total	3178 (50.3)	3134 (49.7)		2551 (50.8)	2472 (49.2)		1419 (59.7)	956 (40.3)		1520 (64.3)	844 (35.7)	
Ever breastfeed												
No	3 (18.8)	13 (81.3)	0.14	6 (50)	6 (50)	0.95	9 (69.2)	4 (30.8)	0.48	24 (49.0)	25 (51.0)	0.024
Yes	3064 (49.6)	3117 (50.4)		2530 (50.9)	2437 (49.1)		1413 (59.7)	954 (40.3)		1499 (64.6)	821 (35.4)	
Total	3067 (49,5)	3130 (50.5)		2536 (50.9)	2443 (49.1)		1422 (59.7)	958 (40.3)		1523 (64.3)	846 (35.7)	
Vaccinated against measles												
No	1332 (56.7)	1017 (43.3)	< 0.01	887 (63.3)	515 (36.7)	<0.01	423 (70.6)	176 (29.4)	< 0.01	363 (80.5)	88 (19.5)	< 0.01
Yes	1720 (45.0)	2099 (55.0)		1663 (46.0)	1953 (54.0)		997 (56.0)	782 (44.0)		596 (61.1)	380 (38.9)	
Total	2052 (49.5)	3116 (50.5)		2250 (50.8)	2468 (49.2)		1420 (59.7)	958 (40.3)		959 (67.2)	468 (32.8)	

In 2001 NDHS survey, babies born into poor families had a 35% higher risk of being stunted than those born in wealthier families (AOR 1.35, CI 95% 1.14–1.60); babies born to families living in mountainous districts had a 45% higher risk of stunting than those born in Terai districts (AOR 1.45, CI 95% 1.12–1.88); and babies who were never breastfed had more than 4-fold risk of stunting than those who had never breastfed (AOR 4.74, CI 95% 1.05–21.27) (Table 2).

**Table 2:** Trends in the risk factors for stunting from 2001 to 2016 in Nepal (NDHS data)

Characteristics	2001	2006	2011	2016
	AOR (95% CI)	AOR (95% CI)	AOR (95% CI)	AOR (95% CI)
Poor family	1.35 (1.14–1.60)	1.04 (0.87–1.23)	1.89 (1.44–2.48)	1.38 (1.05–1.82)
Resides in mountain district	1.45 (1.12–1.88)	1.40 (1.07–1.84)	2.03 (0.92–4.49)	1.01 (0.65–1.57)
Resides in hill district	1.12 (0.96–1.30)	1.30 (1.10–1.53)	1.57(1.00–2.48)	0.95 (0.73–1.24)
Uneducated mother	1.31 (1.11–1.55)	1.49 (1.27–1.75)	1.15 (0.90–1.47)	1.49 (1.17–1.90)
Working mother	0.82 (0.67–1.00)	1.09 (0.89–1.33)	0.99 (0.77–1.29)	0.74 (0.59–0.94)
No toilet facility	1.05 (0.85–1.30)	1.38 (1.16–1.64)	1.10 (0.85–1.41)	1.22 (0.92–1.60)
Cooking fuel	0.88 (0.59–1.31)	0.63 (0.46–0.87)	0.98 (0.69–1.40)	0.83 (0.61–1.12)
Maternal tobacco smoking	1.31 (1.12–1.52)	1.32 (1.10–1.60)	1.20 (0.87–1.64)	1.19 (0.77–1.84)
Antenatal care (< 4 times)	1.31 (1.04–1.65)	1.24 (1.00–1.53)	1.10 (0.86–1.41)	1.29 (1.01–1.66)
No iron supplementation	1.26 (1.05–1.51)	1.12 (0.93–1.36)	1.02 (0.78–1.35)	1.09 (0.75–1.57)
Child delivered at home	1.21 (0.92–1.58)	1.54 (1.24–1.90)	1.82 (1.41–2.35)	1.16 (0.92–1.48)
Never breastfed	4.74 (1.05–21.27)	3.17 (0.71–14.22)	1.68(0.25–11.00)	2.79 (0.85–9.12)
Small size at birth	1.66 (1.42–1.94)	1.56 (1.31–1.87)	1.37(1.13–1.91)	1.32 (1.01–1.73)

*AOR* Adjusted odds ratio, *CI* confidence interval

In the 2006 NDHS survey, babies born at home had 54% higher risk of stunting than those born at a health institution (AOR 1.54, CI 95% 1.24–1.90); babies of uneducated women had 49% higher risk of stunting than babies born to educated women (AOR 1.49, CI 95% 1.27–1.75); and babies who were small size at birth had 56% higher risk of stunting than those who were normal size at birth (AOR 1.56, CI 95% 1.31–1.87) (Table 2).

In the 2011 NDHS survey, babies born at home had 82% higher risk of stunting than those born at a health institution (AOR 1.82, CI 95% 1.41–2.35); babies who were small size at birth had 37% higher risk of stunting than those who were normal size at birth (AOR 1.37, CI 95% 1.13–1.91); and babies born into poor families had a 89% higher risk of being stunted than those born in wealthier families (AOR 1.89, CI 95% 1.44–2.48) (Table 2).

In the 2016, babies who were small size at birth had 32% higher risk of stunting than those who were normal size at birth (AOR 1.32, CI 95% 1.01–1.73); babies born into poor families had a 38% higher risk of being stunted than those born in wealthier families (AOR 1.38, CI 95% 1.05–1.82); and babies of working women had 26% lower risk of stunting than babies of non-working women (AOR 0.74, CI 95% 0.59–0.94) (Table 2).

## Discussion

The nutritional status of young children in Nepal improved between 2001 and 2016. In 2001, the risk of being stunted was higher for babies born in poor families, to non-educated women, who lived in hill or mountain districts, who were small at birth, and whose mothers smoked tobacco, whose mothers did not make any ANC visits, and who did not breastfeed them. The incidence of stunting and the level of risk decreased between 2001 and 2016. Analysis of the 2016 data showed that the mother having paid employment had a protective effect against stunting.

We have explained the association of different determinants of the level of nutrition which is also articulated in Nepal’s second Multi-sectoral Nutrition Plan (2017) (National Planning Commission 2018). The improved access to health services has reduced the risk of stunting that was caused by the lack of antenatal and intrapartum care. However, poor breastfeeding remains a risk for stunting while poverty is still a major determinant for undernutrition and lack of food security. A major reason for the continued influence of poverty on stunting is that conditional cash transfers are not widely implemented in Nepal, although they have been implemented among socially and geographically disadvantaged groups, where good results have been reported (Renzaho et al. 2018). These programs improve nutrition by providing cash incentives to families to improve their children’s diets. Furthermore, maternal education status remains a determining factor for stunting in Nepal, since mothers are the primary caregivers. Maternal education influences mother’s awareness of the importance of diverse diets and good infant and young child feeding practices.

A number of interventions can reduce the risk factors for stunting (Bhutta et al. 2013; Vaivada et al. 2017). The nutrition-specific interventions include the following:

- For mothers—improved adolescent, pre-conception, and maternal health and nutrition, and dietary and micronutrient supplementation.
- For babies and young children—promoting optimum breastfeeding practices; complementary feeding; dietary supplementation; diversification and micronutrient supplementation; the fortification of foods; the treatment of severe acute malnutrition; disease prevention and management; and the provision of nutrition in emergencies (Arifeen et al. 2009; Collins et al. 2006; Debes et al. 2013; Vaidya et al. 2008).

Nutrition-sensitive interventions include improved food production and food security; the institution of social safety nets; improved early child development; maternal mental health care; women’s empowerment; child protection; schooling; water, sanitation, and hygiene; and health and family planning services (Ruel and Alderman 2013).

Poor nutrition is inter-generational and the cycle of the ill effects of malnutrition from one generation to another can take 100 years to end (Martorell and Zongrone 2012). Babies born to under-nourished women will likely be small for their gestational age and carry the risk of being poorly nourished later on. The level of maternal education is associated with empowerment for decision-making and the ability to obtain paid employment, which is a protective factor for good nutrition. The risk of stunting for children residing in hill districts reduced between 2001 and 2016 and was a protective factor in 2016. The prevalence of chronic food insecurity was found to be highest in the Terai belt, where approximately 45% of the population were poor in 2016, thus explaining the widespread poor childhood nutrition in that area (Economic Research and Regional Cooperation Department 2017; Ministry of Agricultural Development 2016; National Planning Commission 2018).

Despite global consensus on the definition of stunting, stunting is often not recognized and not routinely assessed in primary health care settings (de Onis and Branca 2016). Factors associated with growth failure are all generally correlated with poverty which makes it difficult to pinpoint individual factors that predispose the children to stunting in low and middle-income countries (Prentice 2017).

## Limitations

The study had some limitations. First, the study was based on data from four cross-sectional surveys at different time points. A cohort design study may have better explained the behavioural patterns/factors such as breastfeeding. Second, the reported size of babies in the data was based on mothers’ perceptions and so may have not been an accurate measure. Third, poverty was measured through the proxy indicator of the possession of assets rather than purchasing capacity meaning that it may not fully indicate poverty status. Finally, some of the questions asked in the survey might have recall bias, especially on duration of breast feeding to determine exclusive breast feeding.

## Conclusions

The study found that the extent of risk posed by several risk factors for stunting has changed in the last 16 years. Further reducing the extent of childhood stunting requires a multi-sectoral approach especially in terms of further improvements in health service delivery to reduce poor nutrition. Nepal’s government and its development partners need to further invest in maternal and child nutrition not only for the sustainable reduction of child mortality and morbidity but also to ensure the optimal cognitive development of all Nepali children.

## Abbreviation

ANC: Antenatal care
MDG: Millennium development goal
MSNP: Multi-sectoral nutrition plan
NDHS: Nepal demographic health survey
NAGA: Nutritional assessment and gap analysis report
SDG: Sustainable development goal
